# The Week's Work

**Published:** 1911-07-29

**Authors:** 


### The Week's Work.

### LORD IVEAGH AND THE CORK HOSPITALS.

One immediate effect of Lord Iveagh's gift of £15,000 to the Dublin hospitals has been a determination on the part of the North Infirmary, Cork, to bring forward its claims on his generosity. English hospital workers will appreciate the flavour of a decision which exemplifies what we are pleased to call Irish irrationality, for the connection does not seem at first sight a very obvious one. We shall all have, however, enough institutional solidarity to hope that Mr. P. Murphy will be proved right in his assertion that "perhaps Lord Iveagh was only waiting to be approached," and that the North Infirmary may gain sufficient funds to secure the children's ward that Mr. A. M. Cole, J.P., at a special meeting of the board of management, declared to be so sadly needed at present.

### MUTINY MEDAL FOR A HOSPITAL SECRETARY.

Mr. James Briscoe, the secretary of the Bolton Infirmary, has at last received his reward for services rendered in the Indian Mutiny. Mr. Briscoe was then a private in the 72nd Highlanders, which formed part of General Michael's division, was commanded by Colonel Park, and was engaged in field service in Central India. Mr. Briscoe remembers chasing Tantia Topee, the rebel ringleader, and the work of the 72nd largely consisted in protecting the irregular cavalry force of hillmen with which it was associated. The astonishing delay in awarding the medal to Mr. Briscoe has been due, as usual, to military red-tape, which entrenched itself behind a now obsolete order that only men who had been under fire should receive the decoration. As however Mr. Briscoe's regiment was distinguished for putting the enemy under fire so effectually as to escape that contingency itself the application of the rule in this case was hardly a happy one. The result is that last week the medal arrived, accompanied by a letter from Capt. B. S. Stapleton, explaining that it is "granted for service in the suppression of the Indian Mutiny." Need we add that the date of that was 1857-58? The medal bears a clasp with the words "Central India" on a ribbon of red and white. Mr. Briscoe, who declares the end of this long delay in receiving the honour to be due to the help of Mr. A. H. Gill, M.P., already possesses the Crimean, Turkish Crimean, and long-service Volunteer medals. He has held the post of secretary at Bolton Infirmary for at least twenty-five years.

### HOSPITAL BOARDS AND SANATORIUM BENEFIT.

There are signs that the Government's proposal for a special fund of £1,500,000 to be used for making grants for building sanatoria may be used for a somewhat different purpose. The joint hospital boards throughout the country are considering the probability of securing a share of this sum to assist them in paying off their building debts or loans. If they are right in believing that they will receive such grants, the much talked of extension of the sanatoria system and of the "campaign" against tuberculosis will not take place. The local bodies will benefit, and that is all. We mention this point not because we grudge the local authorities their money, but because experience teaches that the payment of old building loans is not the same thing as the extension of a system of buildings. The loans having been undertaken with the sanction of a Government department are, like municipal loans, provided for, and grants in aid of them will possibly gain local support to the giver but will not extend the sanatoria system. This, whether or not it be the best way of dealing with pulmonary tuberculosis, is what the special fund was introduced to do, and it is perhaps worth noting that it may merely be used to subsidise local ratepayers.

### EAST LONDON HOSPITAL FOR CHILDREN.

According to Colonel Charles Needham, the example set by the Queen in suggesting the Coronation gift of £500 from Mr. Sidney Davis has not been followed up by the hospital's subscribers generally. Indeed, though the figures show an increase in the receipts over the corresponding six months of last year, Colonel Needham is reported to have declared the position to be more deplorable than usual. However, the hospital perhaps can comfort itself, as other children's hospitals have been doing, with the expectation that its interests will suffer less than those of institutions for adults, supposing that the Insurance Bill is passed into law, although it would be idle to deny that that contingency is not so certain to-day as its supporters recently would have had us believe.

### NOTIFICATION AT THE BROMPTON.

Apparently the only effect of the Local Government Board's Order for the compulsory notification of cases of consumption in hospitals upon the Brompton Hospital has been the cessation of the voluntary notification which previously prevailed in the institution. This system was discarded on May 1, when the compulsory order came into force. It is noteworthy, however, that the tacit approval of the hospital, which the adoption of its example implies, has not been recognised by its supporters, since during the current year the amount received in legacies is one-fifth only of the average sum received for twenty years from that source. The result has been, of course, a sale of stock and a banker's loan, a hardly satisfactory sequel to its pioneer example.

### COLONEL WARBURTON'S LAST FUNCTION.

The visit of the King and Queen to the Royal Infirmary, Edinburgh, has been the last event of Colonel Warburton's eleven years' service as its medical superintendent, for Sir Joseph Fayrer, his successor, takes up the appointment on Tuesday next, August 1. Their Majesties were awaited by a large number of the managers and of the medical staff, and received by Colonel Warburton and Mr. William S. Caw, J.P., the treasurer and clerk, both of whom had the honour of being presented. Number 7 surgical male ward, which is on the main corridor and was the only unnamed ward in the original building, was visited first and named after himself by the King. The surgical and medical pavilions are connected by a covered corridor with an open verandah, and a visit was paid to Number 30 medical ward for women, which was named by the Queen. The big kitchens were next inspected, where the total number of patients and staff cooked for amounts to over 1,100. On the board of benefactors his Majesty noted the name of George IV., whose name was put down for a contribution of £200, and in the board room an old infirmary class ticket, dated 1768, and belonging to Dr. John Goodsir, was shown. The visitors' book was then signed, whereupon the Lord Provost remarked that the book had been now signed by the King four times and by the Queen three times. A bound copy of the report was presented to their Majesties, and also specimens of the souvenir cards of the Coronation which had been given to every patient on June 22. The Edinburgh Royal Infirmary is, of course, one of the largest hospitals in the United Kingdom.

### HUDDERSFIELD ROYAL INFIRMARY.

Not long ago Mr. John Sykes, J.P., president of the above institution, took steps to secure royal recognition for its work. The local story goes that the borough's member, Mr. Sherwell, was approached, and with his assistance full information as to the infirmary's income, accommodation, and expenditure was forwarded to the King, with the result that Mr. Bate, the secretary of the infirmary, received a letter from the Home Office announcing a favourable answer to the petition. It is worth while repeating once more that the word "Royal" is no magic adjective for increasing a hospital's income. It is a recognition of efforts already made with success, and the process of its acquisition is often a useful stimulus to local subscribers who feel that they have something worth working for in gaining royal acknowledgment. That is why, as at Bolton, a movement to secure it may be supported, even though, if not because, the hospital is in some financial straits.

### THIS WEEK'S DRUG MARKET.

The most noteworthy price alteration that has been made since last week's report appeared is a reduction of 1s. 5d. per ounce in the quotation for cocaine, a reduction which was quite unforeseen. A good business has been done in cascara sagrada at dearer rates. The high price of opium is firmly maintained, but there has been no material advance; morphine and codeine are unchanged in value. Senega is again rather dearer, and oil of peppermint is tending higher in price. Almond oil is slightly dearer. Chamomiles show a dearer tendency. The price of acetic acid has advanced.

